# Vessel-Wall Magnetic Resonance Imaging of Intracranial Atherosclerotic Plaque and Ischemic Stroke: A Systematic Review and Meta-Analysis

**DOI:** 10.3389/fneur.2018.01032

**Published:** 2018-12-03

**Authors:** Han Na Lee, Chang-Woo Ryu, Seong Jong Yun

**Affiliations:** Department of Radiology, Kyung Hee University Hospital at Gangdong, School of Medicine, Kyung Hee University, Seoul, South Korea

**Keywords:** magnetic resonance imaging, intracranial arteriosclerosis, plaque, brain ischemia–diagnosis, cerebrovascular accident, vessel wall imaging, high resolution imaging, systematic (literature) review

## Abstract

**Introduction:** Vessel-wall magnetic resonance imaging (MRI) has been suggested as a valuable tool for assessing intracranial arterial stenosis with additional diagnostic features. However, there is limited conclusive evidence on whether vessel-wall MR imaging of intracranial atherosclerotic plaques provides valuable information for predicting vulnerable lesions. We conducted this systematic review and meta-analysis to evaluate which characteristics of intracranial-plaque on vessel-wall MRI are markers of culprit lesions.

**Methods:** The MEDLINE, EMBASE, and Cochrane Library of Clinical Trials databases were searched for studies reporting the association between vessel-wall MRI characteristics of intracranial plaque and corresponding stroke events. Odds ratios (ORs) for the prevalence of stroke with intracranial-plaque MRI characteristics were pooled in a meta-analysis using a random-effects model.

**Results:** Twenty studies were included in this review. We found a significant association between plaque enhancement (OR, 10.09; 95% CI, 5.38–18.93), positive remodeling (OR, 6.19; 95% CI, 3.22–11.92), and plaque surface irregularity (OR, 3.94; 95% CI, 1.90–8.16) with stroke events. However, no significant difference was found for the presence of eccentricity (OR, 1.22; 95% CI, 0.51–2.91).

**Conclusion:** Based on current evidence, intracranial plaque contrast enhancement, positive remodeling, and plaque irregularity on MRI are associated with increased risk of stroke events. Our findings support the design of future studies on intracranial-plaque MRI and decision making for the management of intracranial atherosclerotic plaques.

## Introduction

During last two decades, a shift has taken place toward imaging for the assessment of atherosclerotic-plaque characteristics rather than the luminal stenosis measurement as a result of accumulating knowledge that the histopathologic composition of plaques is a major risk factor for ischemic symptoms independent of stenosis severity (1). This trend has been broadly applied to assess extracranial carotid stenosis; carotid vessel-wall magnetic resonance imaging (MRI) is emerging as the best candidate for assessing carotid stenosis with additional diagnostic features pertinent to patient management (2, 3).

Intracranial atherosclerotic stenosis (ICAS) is a major cause of ischemic stroke, to a comparable degree with extracranial atherosclerosis, worldwide especially in Asian populations accounting for 10% of transient ischemic attack and 30–50% of ischemic strokes (4, 5). However, vessel-wall imaging in ICAS is somewhat lagging compared to that in extracranial atherosclerosis due to technical limitations in the imaging of small structures and the lack of insight regarding intracranial atherosclerotic plaques. Recently, numerous interesting studies on intracranial-plaque imaging have been published, suggesting that the radiological characteristics of intracranial plaques may be an important predictor of vulnerability in addition to the degree of stenosis (2, 6). Despite recent reports advocating the benefit of intracranial-plaque vessel-wall MRI, there is limited conclusive evidence regarding whether vessel-wall MRI provides valuable information for predicting vulnerable lesions, and which characteristics are useful to judge the vulnerability among many different characteristics other than those previously suggested. Moreover, there is debate on how intracranial plaque morphology is related to the risk of stroke because imaging features had not been proven by histopathological specimens and the assumption of imaging markers has been suggested based on small populations in individual studies, thereby making it challenging to draw definite conclusion on the value of vessel-wall MRI for intracranial-plaque characterization.

Therefore, we considered it necessary to perform a quantitative synthesis of existing evidence to explore fully and present high-level evidence regarding the valuable characteristics of intracranial-plaque vessel-wall MRI. Furthermore, it is worth clarifying whether there are differences in the risk profiles of specific plaque characteristics. Gupta et al. (7) presented a systematic review of high-resolution MRI of intracranial atherosclerotic plaques but they only analyzed one image feature of plaque enhancement. As many pertinent studies have been published since the previous review, a meta-analysis using updated data is needed. In particular, a consensus on MRI characteristics of vulnerable plaques is needed for future prospective studies to determine the clinical benefit of vessel-wall MRI in ICAS and its clinical applications. Summarizing this knowledge might provide guidelines for prospective studies and can also be applied in the clinical field.

For these reasons, we conducted this systematic review and meta-analysis to evaluate whether the characteristics of intracranial-plaque vessel-wall MRI are markers of symptomatic lesions of corresponding ischemic events.

## Methods

### Search Strategy

The meta-analysis was conducted in accordance with the guidelines for meta-analyses of observational studies in epidemiology (8). We conducted a systematic literature review of the PubMed, EMBASE, and Cochrane library databases from inception through June 30, 2018. To identify eligible studies, the following keywords, and entrée terms analogous to these, were used for searching in relevant combinations using the Boolean operators OR and AND: in combination with the words “intracranial atherosclerosis” or “plaque, atherosclerotic” and “stroke” or “brain ischemia,” “magnetic resonance imaging” or “vessel wall imaging” (Supplemental Data: Search Strategy). In addition, we reviewed the reference lists of the included articles and background papers for potentially relevant studies.

### Study Selection

Two researchers reviewed the content of the screened articles for the inclusion criteria listed as follows: studies that (1) enrolled patients with intracranial atherosclerosis, (2) enrolled patients who underwent high-resolution vessel-wall MRI of the intracranial arteries, (3) enrolled more than 10 subjects, and (4) assessed vessel-wall MRI findings of intracranial plaques and their relation to ischemic symptoms. We excluded studies that (1) reported duplicate data, (2) was limited in appropriate data, (3) included non-stenotic lesions (such as the contralateral side of lesions) as controls, and that (4) were markedly flawed with respect to the guidelines of reporting observational studies.

### Data Extraction

Two reviewers independently extracted data from the selected studies that fulfilled the inclusion and exclusion criteria using a standardized form, and all disagreements were resolved by consensus. The following data were collected: report characteristics (first author's name, year of publication, country of the study), major inclusion/exclusion criteria, MRI protocol, basic demographics of subjects, the prevalence of stroke risk factors in the studied populations, including hypertension, diabetes, coronary artery disease, dyslipidemia, and smoking history; the definition of characteristic findings of intracranial plaque, and definitions of culprit and non-culprit lesions.

The quality of the included studies was also independently assessed by two reviewers using the “Quality Assessment Tool for Observational Cohort and Cross-Sectional Studies,” provided by the National Institutes of Health (9).

### Statistical Analysis

Demographic characteristics and extracted covariates are summarized with standard descriptive statistics. Categorical variables are expressed as frequencies, and continuous variables are expressed as means with standard deviations. Continuous variables presented by median and intervals were converted to means and standard deviation (10).

Based on the collected data of the enrolled studies, we assessed the incidence of culprit lesions associated with characteristic plaque findings on vessel-wall MRI. To perform the pooled estimates, these characteristic findings were limited to those present in three studies or more, and included the presence of contrast enhancement, positive remodeling (the ratio of the out-diameter at target lesion to that at reference artery—contralateral or proximal non-stenotic segment- is over 1.05), intraplaque hemorrhage (IPH; high signal intensity on T1-weighted MRI), plaque eccentricity, and irregularity of plaque surface. We defined culprit lesions as intracranial arterial stenosis with (1) corresponding ischemic stroke including transient ischemic attack (TIA) and/or ischemic lesions on MRI or (2) corresponding downstream embolic infarction (large-artery atherosclerosis by the TOAST classification) in the acute or subacute phase. Non-culprit lesions were defined as intracranial arterial stenosis (1) without recent neurologic symptoms relevant to the lesion or (2) with ipsilateral stroke caused by small-vessel occlusion.

Odds ratios (ORs) with corresponding 95% confidence intervals (CIs) were used to determine the associations between ischemic stroke and imaging findings on vessel-wall MRI. The pooled OR for dichotomous parameters was estimated with a random-effect weighted meta-analysis and a forest plot was generated. A continuity correction of 0.5 was applied for studies without event in one arm. Heterogeneity across studies in the meta-analysis was assessed using the I^2^ test, with values higher than 50% considered to indicate substantial heterogeneity. If there was a possibility that the pooled estimates would be confounded by substantial heterogeneity among the studies, the results were not pooled in order to prevent misinterpretation. As for the evaluation of publication bias, it was estimated by contour enhanced funnel plot (11) and Begg's test (12). No publication bias was confirmed when the *p*-value for significance was higher than 0.05.

A subgroup analysis was used to determine whether study-related factors could account for heterogeneity. The subgroup analysis was conducted according to any binary variables that may have affected the consistency of a result across the enrolled studies. In order to assess the robustness of the observed outcomes, we further conducted sensitivity analyses by removing studies with the higher risks of bias, and with the “leave-one-out” method.

Meta-regression analysis investigated potential effects of hypertension, diabetes mellitus, dyslipidemia, current smoking status, and coronary ischemic disease on intracranial MRI characteristics associated with culprit lesions.

## Results

### Study Selection

**Figure 1:**
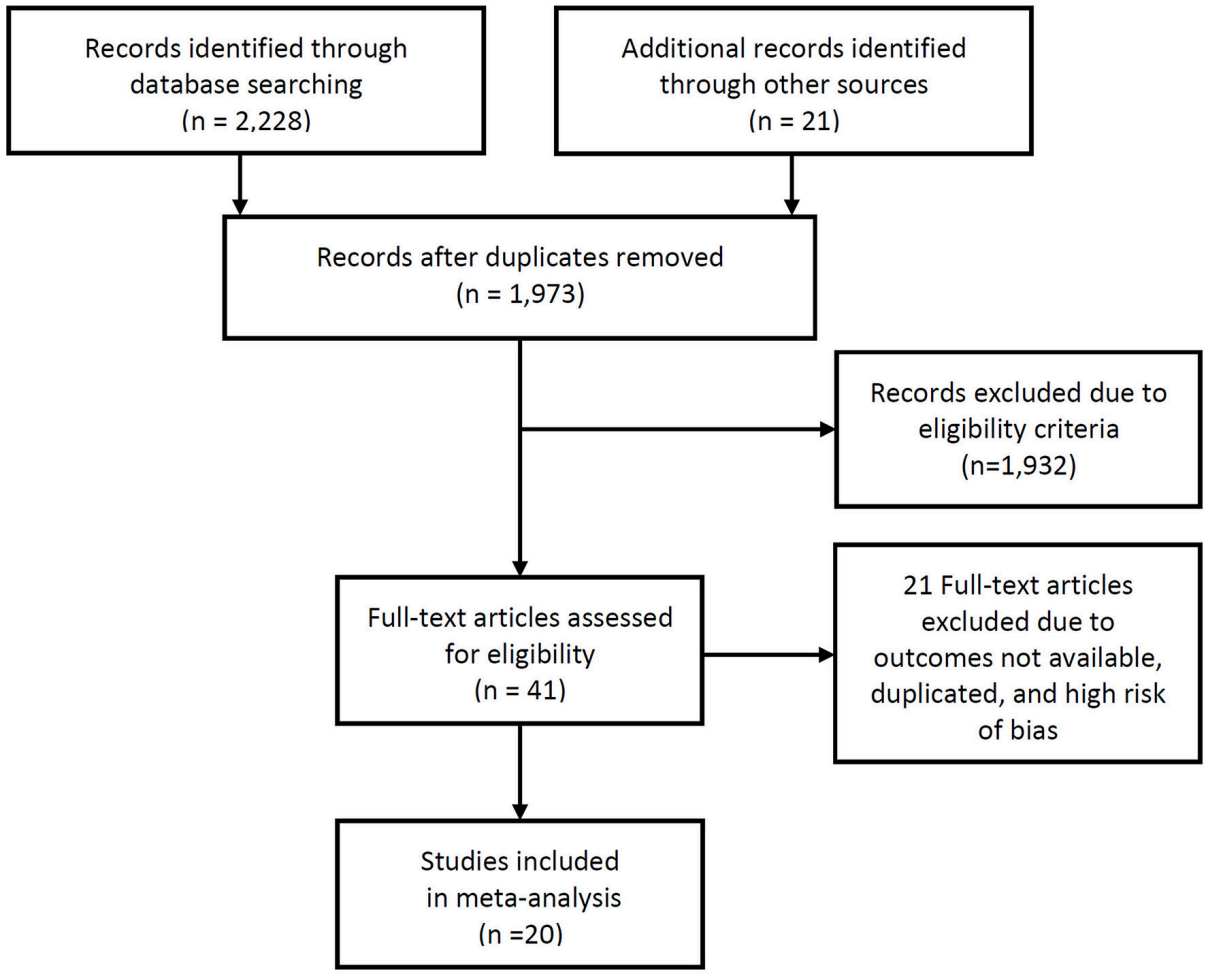
PRISMA flow diagram of studies identification.

A total of 1,973 studies were identified during the initial search. Of these, review of the titles and abstracts identified 41 studies that presented the stroke rate and intracranial-plaque vessel-wall MRI findings. After reviewing the full texts, 20 studies were suitable for inclusion to this review (2, 6, 13–30). Two studies (2, 18) that were published from our institute were finally included in the current analysis, and these data were reanalyzed based on raw data including clinical records and images to obtain the necessary information to conduct the meta-analysis. Since we did not only review the published manuscripts but also retrospectively reanalyzed clinical data, we obtained the approval of the institutional review board and the requirement for informed consent was waived due to the retrospective nature of the study. A flow-diagram summarizing the literature search is presented as Figure 1.

### Study Characteristics

The results of the quality assessment were satisfactory with all the studies satisfying at least 10 of the 14 domains. All studies showed a fatal flaw concerning the sample size justification. Eleven of twenty studies showed a high risk of bias by potential confounders which were not adjusted by logistic regression of other regression methods (2, 13–16, 19, 21–23, 25, 27) (Supplemental Data: Table).

The basic demographics and the prevalence of risk factors were summarized in Table 1. In total, A total of 1,233 intracranial stenosis lesions of 1,126 patients were eligible for the meta-analysis. In all studies, vessel-wall MRI was prospectively acquired from ICAS or stroke cohorts and was analyzed retrospectively. Fourteen (2, 6, 13–16, 18, 19, 22, 24, 25, 27, 28, 30) and three studies (23, 26, 29) enrolled only middle cerebral artery (MCA) plaques (one study including 7% internal carotid artery (ICA) plaques was enrolled) and only basilar artery (BA) plaques in accordance with their inclusion criteria, respectively, and three studies (17, 20, 21) enrolled both large- and medium-size intracranial arteries for their subjects. The proportion of MCA lesions and BA lesions to total lesions were 74.1%, 25.9%, respectively (Table 1).

**Table 1:** Demographics and risk factor of enrolled studies.

**References**	**Country**	**Subject No^a^**	**Mean age (years), SD**	**Male (%)**	**Lesion site**	**HTN (%)**	**DM (%)**	**Dyslipidemia (%)**	**Current smoking (%)**	**CAD (%)**
Ryu et al. (2)	South Korea	16(14)	60.0 ± 8.5	57.1	MCA	78.6	50.0	50.0	14.3	7.1
Xu et al. (13)	China	61	62.4 ± 11.6	63.9	MCA	68.9	29.5	31.1	36.1	N/A
Chung et al. (14)	South Korea	30	65.8 ± 9.7	63.3	MCA	56.7	46.7	70.0	43.3	N/A
Kim et al. (15)	South Korea	26	65.2 ± 10.5	61.5	MCA	65.4	30.8	23.1	NA	23
W Xu et al. (16)	China	109(104)	56.7 ± 12.8	83.7	MCA	69.2	23.1	46.2	21.2	N/A
Vakil et al. (17)	United States	22(19)	68.7 ± 9.6	68.4	ICA, A2, BA, V4	89.5	47.4	84.2	10.5	N/A
Ryu et al. (18)	South Korea	36	69.7 ± 11.9	47.2	MCA	69.4	44.4	63.9	33.3	11.1
Yang et al. (19)	China	65(73)	63.0 ± 11.3	63.0	MCA	N/A	N/A	N/A	N/A	N/A
Qiao et al. (20)	United States	99(27)	56.8 ± 12.4	70.4	M1-2, A1-2, C3-4, P1-2, BA, V4	81.5	33.3	59.3	14.8	N/A
Ryoo et al. (21)	South Korea	80	64.5 ± 14.8	73.8	MCA (75.3%) BA (24.7%)	67.5	41.3	51.3	27.5	11.3
P Xu et al. (22)	China	32	65.8 ± 13.1	46.9	MCA	78.1	28.1	59.4	34.4	37.5
Yu et al. (23)	South Korea	73	72.4 ± 55.8	56.2	BA	46.6	27.4	34.2	23.3	16.4
Zhao et al. (6)	China	51	67.4 ± 8.8	58.8	MCA	64.7	35.3	N/A	31.4	N/A
Teng et al. (24)	China	165(139)	57.1±?	64.7	MCA	71.2	34.5	N/A	29.5	9.4
Zhang et al. (25)	China	33	68.1 ± 11.8	81.8	MCA	81.8	51.5	N/A	57.6	N/A
Wang et al. (26)	China	57	59.4 ± 8.1	77.2	BA	63.2	52.6	21.1	49.1	N/A
Jang et al. (27)	South Korea	25	62.7 ± 6.19	40.0	MCA	48.0	76.0	80.0	28.0	N/A
Wu et al. (28)	China	74	54.7 ± 12.1	79.7	MCA (92%) ICA (8%)	75.7	21.6	40.5	52.7	N/A
Zhu et al. (29)	China	126	61.5 ± 10.0	65.1	BA	80.2	33.3	51.6	27.8	4.0
Lu et al. (30)	China	46	55.8 ± 15.2	67.4	MCA	76.1	28.3	47.8	28.3	N/A

*SD, standard deviation; HTN, hypertension; DM, diabetes mellitus; CAD, coronary artery disease; MCA, middle cerebral artery; ICA, internal carotid artery, A2, anterior cerebral artery segment 2; BA, basilar artery; V4, vertebral artery segment 4; M1-2; middle cerebral artery segment 1-2; A1-2, anterior cerebral artery segment 1-2; C3-4, cavernous and supraclinoid segments of internal carotid artery; P1-2, posterior cerebral artery segment 1-2; N/A, data not available*.

a *Data indicates numbers of analyzed lesions with numbers of patients in parentheses*.

In all studies, target lesions for vessel-wall MRI were assessed by MR angiography (MRA) before the acquisition of MRI, and 11 of 20 studies (13, 14, 16–20, 22, 23, 26, 30) enrolled only patients with moderate to severe stenosis. Although 14 studies (2, 6, 13, 14, 16–19, 22, 23, 25, 26, 29, 30) recruited subjects based on only stenotic lesions on angiographic imaging, six studies (15, 20, 21, 24, 27, 28) recruited subjects based on ischemic symptoms. Four of these six studies (15, 21, 27, 28) dichotomized subjects into infarction by large-artery atherosclerosis (downstream embolic infarction) and small-vessel occlusion according to the subtype of ischemic stroke at the ipsilateral side of the stenotic lesion (Table 2).

**Table 2:** Inclusion/exclusion criteria, definition of ischemic event, and MRI protocols of enrolled studies.

**References**	**Inclusion criteria**		**Exclusion criteria**			**Definition of culprit plaque**	**MRI protocols**						
	**Patient selection**	**Vessel stenosis degree and modality**	**Cardiac embolism**	**Extra- LAO**	**Non-atheroscl-erosis^a^**	**Culprit lesion, ipsilateral**	**Interval between symptom to MRI**	**MRI field strength (tesla)**	**High-resolution MRI sequences**	**T1-weighted imaging**			
										**Sequences**	**TR/TE (ms)**	**Voxel size (mm^3^)**	**NEX**
Ryu et al. (2)	Lesion	Any stenosis	O	O	O	Ischemic symptoms	1 week	3	T1W, T2W, PD	2D, BB, TSE	581/20	0.38 × 0.48 × 2	4
Xu et al. (13)	Lesion	>50% MRA	O	O	O	Ischemic symptoms	4 week	3	T1W, T2W	2D	800/12	0.25 × 0.25 × 2(ZFIP)	4
Chung et al. (14)	Lesion	>50% MRA	O	O	O	Ischemic symptoms and DWI restriction	N/A	3	T1W, T2W, PD	2D, BB, TSE	700/23	0.39 × 0.61 × 2	4
Kim et al. (15)	Symptom	N/A	O	O	O	Downstream lesion^b^	1 week	3	T1W, T2W, PD	2D, BB,	600/12	0.31 × 0.45 × 2	4
W Xu et al. (16)	Lesion	>70% MRA	O	O	O	DWI restriction	4 week	3	T1W, T2W	2D	800/12 520/12.2	0.25 × 0.25 × 2(ZFIP)	4
Vakil et al. (17)	Lesion	>70% MRA	N/A	O	N/A	Ischemic symptoms and DWI restriction	1day^c^	1.5	T1W	2D, spin echo	63/15	0.9 × 0.9 × 5	N/A
Ryu et al. (18)	Lesion	>70% MRA	O	O	O	Lesion on MRI	3days	3	T1W	3D, BB, VISTA	350/19.5	0.34 × 0.34 × 1	N/A
Yang et al. (19)	Lesion	>50% CA	O	O	O	Ischemic symptoms and DWI restriction	17days	3	T1W, T2W	2D, BB, FSE	1200-2000/50	0.5 × 0.5 × 2	4
Qiao et al. (20)	Lesion and symptom	>50% MRA, CTA, CA	O	O	O	Ischemic symptoms and lesion on MRI	21days^d^	3	T1W	3D, BB, VISTA	2000/38	0.4 × 0.4 × 0.4	1
Ryoo et al. (21)	Symptom	Any stenosis	O	O	O	Downstream lesion^b^	1 week	3	T1W, T2W, PD, FLAIR	3D, BB, VISTA	2100/10	0.25 × 0.25 × 2(ZFIP)	2
P Xu et al. (22)	Lesion	>50% MRA	N/A	N/A	O	DWI restriction	3days	3	T1W, T2W, PD	2D	600/12	0.22 × 0.22 × 2.5	4
Yu et al. (23)	Lesion	>50% MRA	N/A	O	O	Ischemic symptoms	2 week	3	T1W, T2W	2D, BB, TSE 3D, BB, MPRAGE	800/10	0.65 × 0.71 × 2	2
Zhao et al. (6)	Lesion	>30% MRA	O	O	O	Ischemic symptoms	1 week	3	T1W, T2W, PD	2D, BB T1W 3D, SPACE	700/26	0.55 × 0.55 × 2	2
Teng et al. (24)	Lesion and symptom	Any stenosis	O	O	O	Ischemic symptoms and lesion on MRI	3.3days^d^	3	T1W, T2W	2D, BB, FSE	567/16	0.31 × 0.39 × 2	2
Zhang et al. (25)	Lesion	>30% MRA	O	O	O	DWI restriction	1 week	3	T1W, T2W, PD	2D, BB	1000/9	0.44 × 0.56 × 2	2
Wang et al. (26)	Lesion	>50% MRA	O	N/A	O	Lesion on MRI	4 week	3	T1W	3D, BB, SPACE	900/15	0.5 × 0.5 × 0.5	N/A
Jang et al. (27)	Symptom	N/A	O	O	O	Downstream lesion^b^	3days	3	T1W	3D, BB, SPACE	700/12	0.9 × 0.9 × 0.9	1
Wu et al. (28)	Symptom	Any stenosis	O	O	O	Downstream lesion^b^	2 week	3	T1W	3D, SPACE	900/15	0.5 × 0.5 × 0.5	N/A
Zhu et al. (29)	Lesion	>30% MRA, CTA, CA	O	N/A	O	Ischemic symptoms	12 week	3	T1W, T2W	2D, FSE	601/10	0.4 × 0.4 × 2	N/A
Lu et al. (30)	Lesion	>50% MRA	O	O	O	Ischemic symptoms and DWI restriction	1 week	3	T1W	3D, BB, SPACE	900/4.2	0.4 × 0.4 × 0.75	N/A

*Extra-LAO, stenosis at extracranial carotid artery; TR, repetition time; TE, echo time; NEX, number of excitations; MRA, magnetic resonance angiography; CA, cerebral angiography; CTA, computed tomography angiography; DWI, diffusion-weighted magnetic resonance imaging; T1W, T1-weighted; T2W, T2-weighted; PD, proton density; FLAIR, fluid-attenuated inversion recovery; 2D, two dimensional; 3D, three dimensional; BB, black blood; TSE, turbo spin echo; VISTA, volume isotropic turbo spin echo acquisition; FSE, fast spin echo; MPRAGE, magnetization prepared rapid gradient echo; SPACE, sampling perfection with application optimized contrasts using different flip angle evolution; ZFIP, zero filled interpolation; N/A, data not available*.

a *Nonatherosclerosis vasculopathy such as dissection, vasculitis or Moyamoya disease*.

b *Downstream of culprit lesion indicates artery-to-artery embolic infarctions caused by intracranial atherosclerosis and corresponding non-culprit lesion means ipsilateral infarction caused by small-vessel occlusion*.

c *Data is limited to 16 of 19 patients*.

d *Data is median value of interval between onset of symptom to MRI scan*.

Most enrolled studies used 3.0 Tesla MRI units to acquire vessel wall MRI, acquired with sub-mm in-plane voxel resolution, lower than 0.6 × 0.6 mm. Intracranial plaques were assessed by high-resolution, multi-contrast vessel-wall MRI in 13 studies, of which seven studies (2, 6, 14, 15, 21, 22, 25) used three different sequences of T1-,T2- and proton density-weighted images and six studies (13, 16, 19, 23, 24, 29) included T1- and T2-weighted images. All studies included T1-weighted MRI with 14 studies using different block-blood techniques; six studies (2, 14, 15, 19, 24, 25) used 2-dimensional turbo spin echo or fast spin echo images; eight studies (6, 18, 20, 21, 23, 26, 27, 30) used 3-dimensional volume isotropic turbo spin echo acquisition or magnetization prepared rapid gradient echo or sampling perfection with application optimized contrasts using different flip angle evolution. All studies except one (20) involved more than one reader evaluating the vessel-wall MRI for ICAS (Table 2).

Stroke events were present in 11 studies evaluating the contrast enhancement of plaques. Six of 11 studies (15, 17, 18, 21, 22, 26) classified the degree of contrast enhancement as a two-level grading system (non-enhancement and enhancement) and five studies (20, 26–28, 30) classified it as a three-level grading system (0: enhancement was less than or equal to that of intracranial arterial walls without plaque, 1: less enhancement than the pituitary stalk, 2: enhancement greater than or equal to that of the pituitary stalk). To calculate the OR by binary group, we dichotomized the three-level grading system as grade 0 and grade 1 to 2.

### Data Synthesis and Statistical Analysis

**Figure 2:**
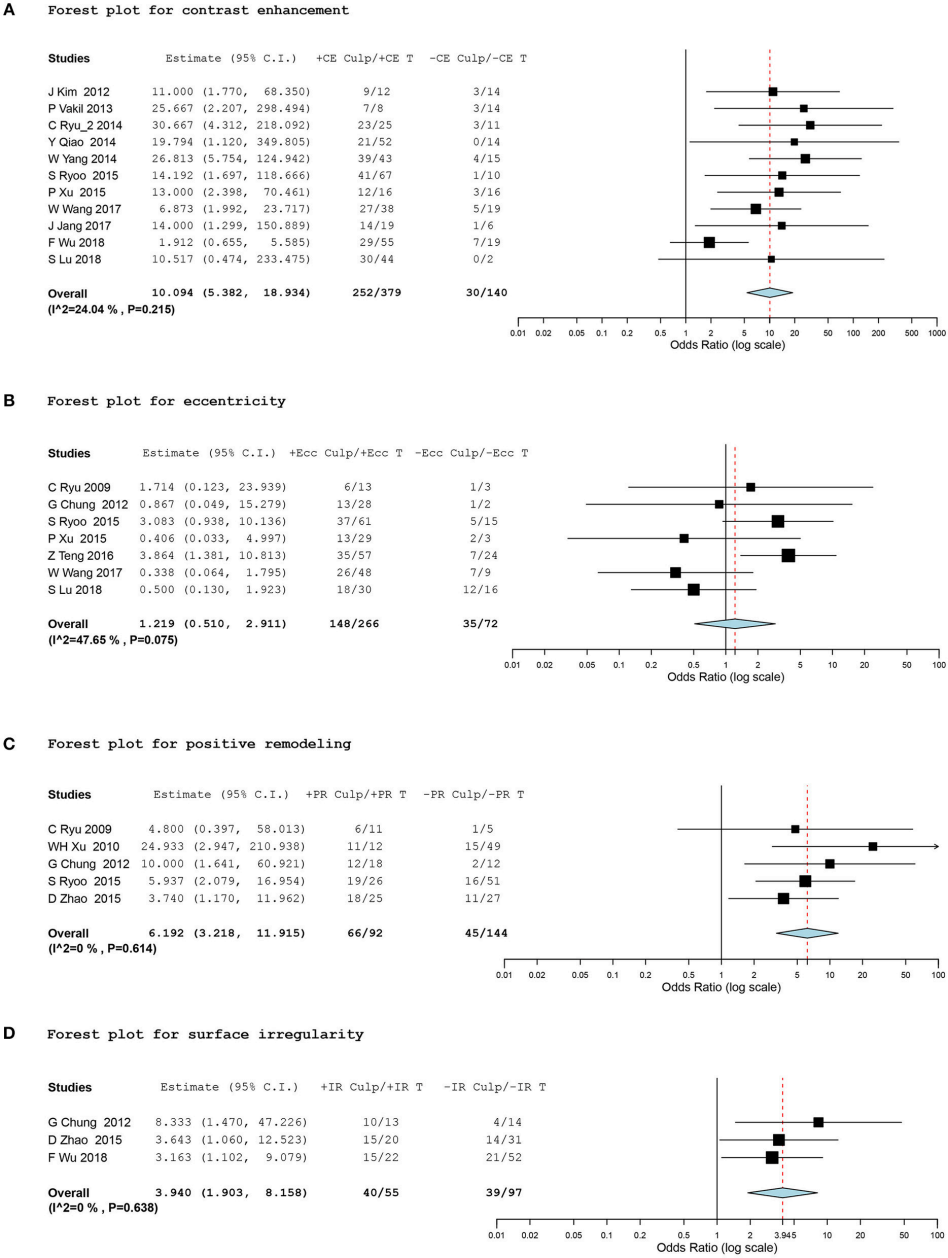
Forest plots for association between vessel-wall MRI findings and ischemic event. Forest plots showing odds ratio (OR) presenting corresponding ischemic events of intracranial atherosclerotic plaques in comparison of positive and negative culprit signs on vessel-wall MRI. The size of the black box corresponding to each study is proportional to the sample size. The horizontal line shows the corresponding 95% confidence interval (CI) of the effect size (OR). The combined estimate is based on a randomized-effects model shown by the diamond. “Culp” and “T” indicate the number of culprit lesions and total lesions according to positive and negative signs of contrast enhancement (CE), eccentricity (Ecc), positive remodeling (PR), and surface irregularity (IR). •[(A)] Forest plot for contrast enhancement •[(B)] Forest plot for eccentricity •[(C)] Forest plot for positive remodeling •[(D)] Forest plot for surface irregularity.

In terms of plaque contrast enhancement, 519 intracranial atherosclerotic lesions in 11 studies (15, 17–22, 26–28, 30) provided data eligible for the meta-analysis. We found a significantly higher prevalence of stroke events in plaques with contrast enhancement, with a random effect OR of 10.09 (95% CI, 5.38 to 18.93; I^2^ = 24.04%; Figure 2A).

Subgroup analysis was conducted for binary classifications according to (1) selecting indication for plaque location (MCA only vs. other intracranial arteries with/without the MCA), (2) the classifying method of culprit and non-culprit lesions (based on the presence of corresponding ischemic symptoms vs. based on TOAST subgrouping of ischemic stroke, large artery embolism and small vessel occlusion), and (3) the grading method of the degree of contrast enhancement (two- vs. three-level grading system). Pooled estimates showed a consistent strong positive correlation between ischemic stroke and plaque contrast enhancement regardless of subgrouping (Table 3).

**Table 3:** Results of subgroup analyses of contrast enhancement of plaque for prediction of ischemic stroke.

**Category**	**Subgroup**	**Studies no**.	**Odds ratio (95% CI)**	**I^2^ (%)**
Plaque location^a^	MCA only^b^	7	10.44(4.06–26.88)	48.7
	Other site^c^	4	10.65(4.21–26.93)	0
Classification of culprit and non-culprit	Ipsilateral stroke	7	14.70(7.34–29.43)	0
	TOAST subgroup^d^	4	6.12(1.89–19.86)	44.1
Degree of contrast enhancement	Two grading	6	18.55(8.65–39.76)	0
	Three grading	5	5.13(2.12–12.41)	21.3

*CI, confidence interval; MCA, middle cerebral artery; ICA, intracranial artery; TOAST, trial of Org 10172 in acute stroke treatment*.

a *Refer to 6th column of Table 1*.

b *Data that evaluated 92% of middle cerebral artery and 8% of internal carotid artery by Wu et al. (28) was also included*.

c *Other intracranial arteries with or without middle cerebral artery*.

d *Culprit lesion was defined as intracranial arterial stenosis with downstream embolic infarction caused by large-artery atherosclerosis and non-culprit lesion was defined as that with ipsilateral stroke caused by small-vessel occlusion (refer to 7th column of Table 2)*.

**Figure 3:**
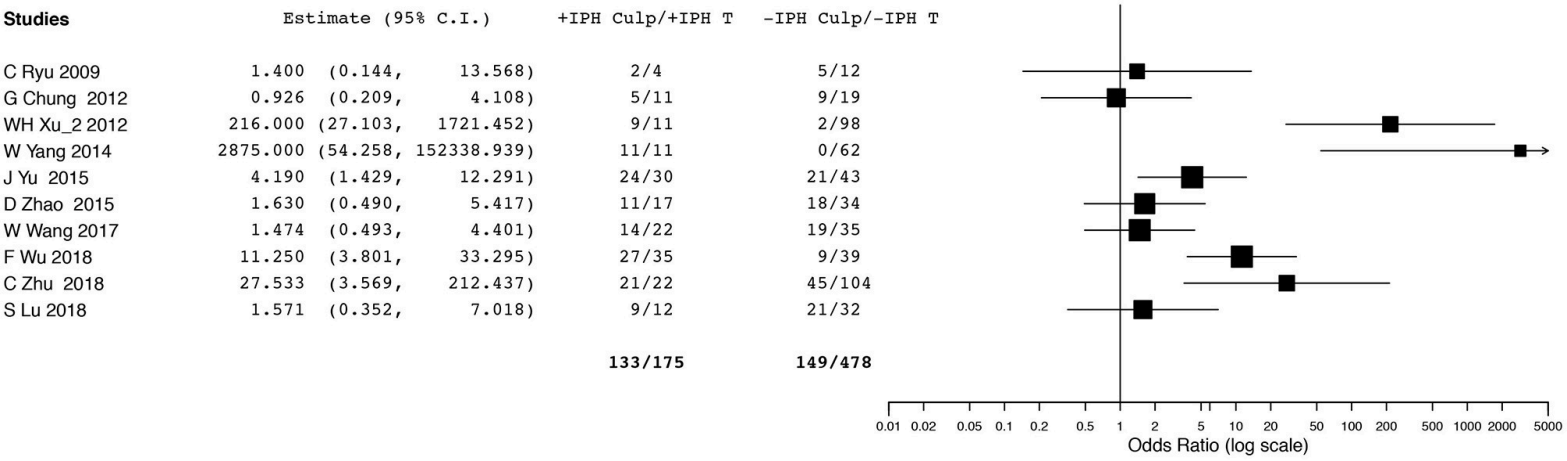
Forest plot for association between intraplaque hemorrhage and ischemic event. Odd ratios and 95% confidence intervals demonstrating association between ischemic event and intraplaque hemorrhage in each enrolled study.

Ten studies (2, 6, 14, 16, 19, 23, 26, 28–30) evaluating IPH reported data that could be included in the meta-analysis. However, we did not conduct OR pooled estimate because we observed significant heterogeneity in the analysis. Although five (16, 19, 23, 28, 29) of 10 studies presented higher prevalence of culprit lesions for positive IPH than for negative IPH, the other five (2, 6, 14, 26, 30) did not find a significant difference in culprit lesions related to IPH (Figure 3).

A total of 338 lesions in seven studies (2, 14, 21, 22, 24, 26, 30), 235 lesions in five studies (2, 6, 13, 14, 21), and 152 lesions in three studies (6, 14, 28) were meta-analyzed for eccentricity, positive remodeling, and plaque irregularity, respectively. The meta-analysis did not show any significant differences in ischemic events between the eccentricity and concentricity of stenosis (OR, 1.22; 95% CI, 0.51 to 2.91; I^2^ = 47.65%). However, we found a significant association between positive remodeling and plaque irregularity and stroke events within the corresponding vascular territory, with a random effect OR of 6.19 (95% CI, 3.22 to 11.92; I^2^ = 0%) and 3.94 (95% CI, 1.90 to 8.16; I^2^ = 0%), respectively (Figures 2B–D).

### Sensitivity Analysis and Meta-Regression

**Figure 4:**
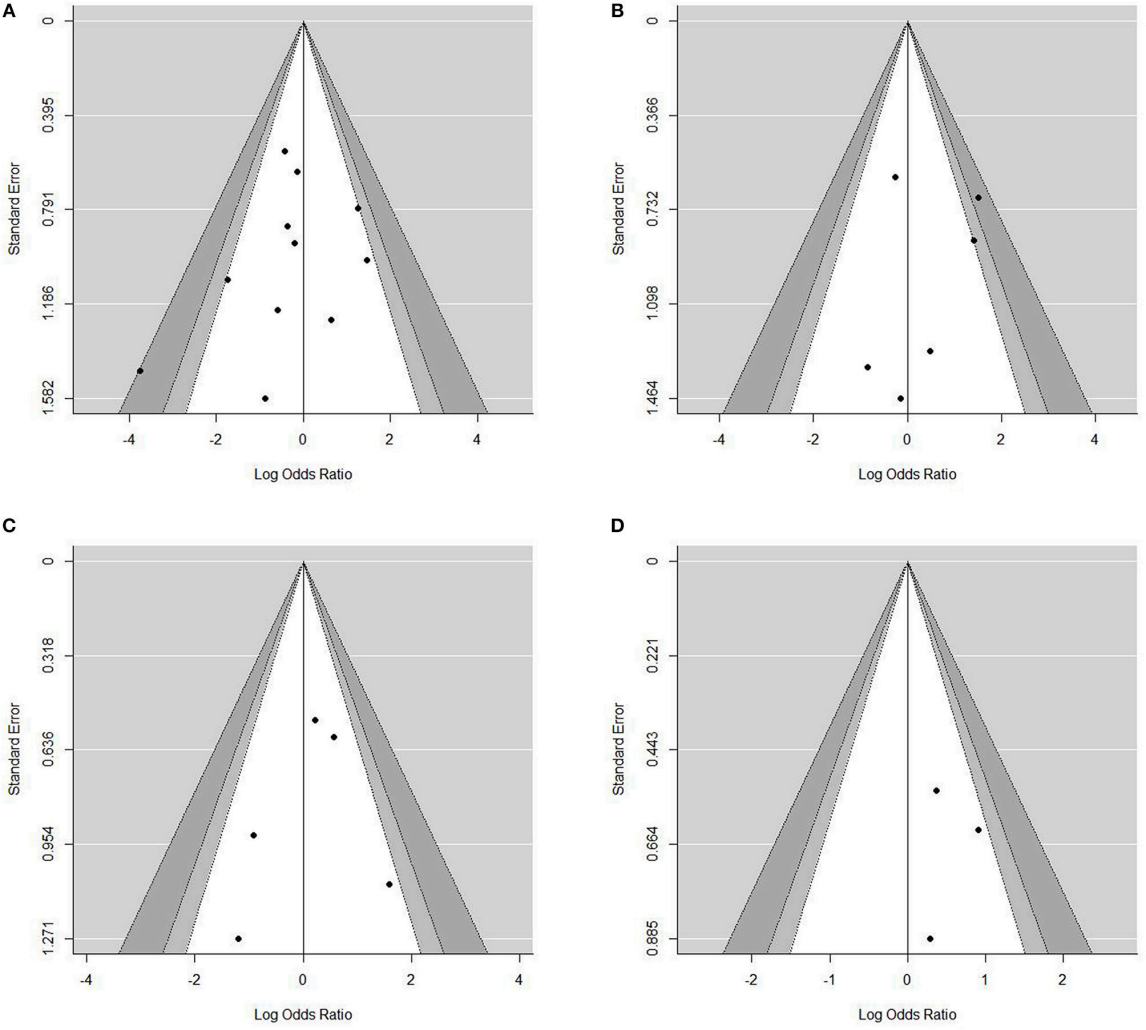
Contour-enhanced funnel plots for the assessment of publication bias. Dots represent point estimates plotted over standard error. Shaded areas represent a given level of significances (*p*-values of 0.01, 0.05, 0.1). •[(A)]Funnel plot for contrast enhancement. •[(B)] Funnel plot for eccentricity •[(C)] Funnel plot for positive remodeling •[(D)] Funnel plot for surface irregularity.

Sensitivity analysis for studies with high risk of bias at (1) study population definition (2) exposure measurement and (3) outcome measurement, and the leave-one-out method showed that the conclusions were not drastically changed with these analyses. Contour enhanced funnel plots indicated that all studies were within the non-significant area (Figure 4) and significant publication bias was not observed based on Begg's test (Table 4) in any meta-analysis.

**Table 4:** Publication bias measures.

**Characteristics of plaque on MRI**	**Publication bias (begg and mazumdar rank correlation)**		
	**Kendell τ**	**Z-value for τ**	***P*-value**
Contrast enhancement	0.109	0.467	0.640
Eccentricity	−0.190	0.601	0.548
Positive remodeling	0.100	0.245	0.807
Irregularity	0.667	1.044	0.296

Meta-regressions were conducted only for studies evaluating plaque enhancement. Information regarding dyslipidemia was available in nine studies. Meta-regression showed a statistically significant negative association between the proportion of current smoking and culprit lesions (slope coefficient (standard error) = −0.062 (0.023), *p* = 0.0006). Information on hypertension, diabetes, and dyslipidemia were available in 10 studies, and meta-regression for these risk factors showed no statistically significant association between these proportion of risk factors and ORs of association between ischemic event and plaque enhancement.

## Discussion

Our meta-analysis, by pooling the available evidence, indicated that intracranial plaques with contrast enhancement, positive remodeling, and wall irregularity are significantly more likely to be associated with ischemic stroke at the corresponding territory. These findings were significantly different from the known vulnerability markers (including IPH, large lipid core, and thin fibrous cap) in the extracranial carotid plaque, suggesting that unlike plaque imaging of the extracranial carotid artery, an independent imaging protocol should be provided to assess the intracranial plaque vulnerability, although intracranial plaque MRI studies have been benchmarked based on carotid plaque studies.

The current results identified vulnerable imaging markers for ICAS, and these could be used as a theoretical foundation for future research assessing the clinical benefit of intracranial vessel-wall MRI, such as monitoring of patients with ICAS or of their response to therapeutic intervention.

Additionally, our study illustrates important limitations of the current literature on MRI plaque characterization. Although there were many candidates for this review, only a limited number of studies were enrolled due to heterogeneity in methodology and selection indication for subjects. This review highlighted the need for more data to confirm and refine the standardization of methods assessing intracranial plaques using vessel-wall MRI.

### Contrast Enhancement

Our meta-analysis revealed the strong association between plaque contrast enhancement and recent ischemic events. Although in line with the result of a previous meta-analysis that presented a pooled OR of 10.8 (95% CI 4.1–28.1) (7), our results differed from those of the previous study in many respects. One third of the studies we included were published after the previous meta-analysis. The relationship between contrast enhancement and ischemic symptoms was adjusted using subgroup analysis for vascular anatomy, grading method of contrast enhancement, and definition of culprit lesion. We excluded two studies that were included in the previous meta-analysis due to selection bias.

As we demonstrated in our study, intracranial plaque enhancement has been known as an imaging marker for plaque vulnerability, and some studies have suggested that this enhancement can be independent of stenosis degree; its mechanism can be explained by vessel-wall neovascularization and inflammation (31). Although contrast enhancement also has been recognized as MRI sign of plaque vulnerability in extracranial carotid arteries, it serves as less precise marker of vulnerable plaque than IPH or thin fibrous cap showing on non-enhanced multi-contrast MRI. However, the present meta-analysis suggested that plaque enhancement might be highly reliable to predict high-risk plaques in intracranial arteries.

This meta-analysis also found that plaque enhancement was more significantly related to infarction caused by large-artery atherosclerosis than to infarction caused by small-vessel occlusion. This finding strengthened the assumption that plaque enhancement can help discriminate the subtype of stroke in ipsilateral stenotic lesions (27).

### Arterial Remodeling

Arterial remodeling is an important mechanism in the pathogenesis of atherosclerosis and this mechanism has been explored in the carotid and coronary arteries (32, 33). Vascular remodeling is related to plaque area and plaque components, supporting higher vulnerability in positive remodeling than negative remodeling with the same degree of stenosis (34). The present meta-analysis confirmed that positive remodeling can be a specific marker of vulnerability in intracranial plaques, in line with previous suggestions regarding the coronary arteries.

In our experience, there are several limitations in effective acquisition of vessel-wall MRI to monitor intracranial remodeling. It can be used for relatively larger-size arteries to obtain adequate resolution due to marginal blurring. It is necessary to identify the target lesion when obtaining the cross-sectional image.

### IPH

A meta-analysis that analyzed longitudinal observational studies on extracranial carotid plaques with MRI found that the presence of IPH is a reliable predictor of subsequent stroke or transient ischemic attack (35). However, half of the enrolled studies in the current review did not report a statistically significant correlation between IPH and recent ischemic stroke, and we could not sum the OR of each study due to significant heterogeneity. A major limitation of IPH in intracranial plaques is that we cannot be certain whether high signal intensity within plaque on T1 weighted MRI is true IPH, because there has been no direct histological evidence for IPH on plaque MRI imaging. Another limitation of IPH detection through plaque vulnerability is the low prevalence of IPH at the site of the stenosis (16), suggesting low negative predictability. In carotid plaque imaging, multi-contrast MRI composed of four or more sequences (T1, T2, proton-density-weighted, the source images of time of flight MR angiography, and others) is recommended to delineate major imaging markers of vulnerability including IPH, lipid-rich necrotic core, and rupture of the fibrous cap. However, the present results suggested that pre-and post-contrast T1-weighted MRI and/or proton density MRI is sufficient to show major high-risk findings, contrast enhancement, degree of remodeling, and plaque irregularity.

## Limitations

Our study illustrates several limitations of MRI plaque characterization related to ischemic stroke risk. First, all included studies had small sample sizes, with limited power for subgroup analyses. The pooled results of our meta-analysis need to be considered in the context of the included studies, in which the number of subjects in some of the subgroups was low. Second, the included studies employed different methodologies. There was variation in study design, eligibility of patient inclusion, and reporting outcomes. Although the statistical analysis of heterogeneity in effect sizes showed homogeneity among studies, the methodological diversity may have led to misinterpretation of the pooled estimates. Third, we could not adjust our estimates for the potentially confounding factors of plaque volume or degree of stenosis severity, which were not systematically specified in all included studies. This lack of data reduced the study's power and decreased estimate precision. In this study, we attempted to reduce the heterogeneity by presenting the results of subgroup analysis in patients with MCA lesions. The predictability of each finding may be meaningful, but more useful findings could not be determined.

## Conclusion

In this study, by pooling the available evidence, we identified three imaging markers of culprit lesions for intracranial plaques: contrast enhancement, positive remodeling, and plaque irregularity, and these can lead to improved patient diagnosis and better decision-making for clinicians. Recently, the appearance of studies regarding intracranial vessel-wall MRI has been rapidly increasing in the literature, but they involve limitations in terms of research quality.

No obvious clinical benefit has been obtained from the use of intracranial vessel-wall MRI, since few of the published studies had a prospective design or involved controlled comparisons. Based on the present study, it is necessary to strengthen the theoretical basis for future prospective studies by developing a method of morphologic-characteristic description and standardization of imaging parameters of intracranial plaques.

## Author Contributions

C-WR conceived and designed the study, collected data, provided guidance, oversight of the study and manuscript, and drafted the manuscript. HNL collected data, performed the statistical analysis, and contributed to manuscript drafting. SJY collected data and performed the statistical analysis.

### Conflict of Interest Statement

The authors declare that the research was conducted in the absence of any commercial or financial relationships that could be construed as a potential conflict of interest. The reviewer HSK declared a past co-authorship with one of the authors C-WR to the handling editor.
