# Supplementary material for: Vessel-Wall Magnetic Resonance Imaging of Intracranial Atherosclerotic Plaque and Ischemic Stroke: A Systematic Review and Meta-Analysis
Source: Front Neurol. 2018 Dec 3;9:1032. doi: 10.3389/fneur.2018.01032 (PMC6287366; doi:10.3389/fneur.2018.01032)
Supplement: Supplementary file 1 [file Data_Sheet_1.docx]

**Supplemental Data**

**I. Search Strategy**

**II. Supplemental Table**

**I. Search Strategy**

Searches were undertaken by using the following specific search queries for electronic databases, without language restriction. We considered all publications included from the earliest available year of online indexing up to June 31 2018.

**Search Query for Pubmed**

1. Stroke[Mesh]

2. Stroke*[tw]

3. cerebrovascular[tw]

4. “Brain Ischemia”[mesh]

5. ((brain OR vascular OR lacunar OR venous OR cerebral OR ischemi*) AND (accident* OR infarct* OR event* OR attack*))

6. CVA[tw] OR CVAS[tw]

7. “Magnetic Resonance Imaging”[mesh]

8. “magnetic resonance”[tw] OR MR[tw] OR MRI[tw] OR MRIs[tw]

9 “contrast media”[Mesh]

10. (Gadolinium or Gd?DTPA or Magnograf or Magnevist or gadopentetate)[tw]

11. “high-resolution*”[tw] OR “high resolution*”[tw]

12. “vessel wall”[tw] OR “vessel-wall”[tw]

13. “plaque imag*”

14. “Intracranial Arteriosclerosis”[mesh]

15. “Plaque, Atherosclerotic”[mesh]

16. intracranial OR cereb* OR brain

17. LRNC OR athero* OR stenos* OR occlus* OR ulcer* OR plaque* OR intraplaque OR hemorrhage* OR IPH OR OR constrict* OR bruit* OR lipid OR fibrous OR culprit OR lesion*

18. OR/1-6

19. OR/7-10

20. OR/11-13

21. OR/14-17

22. AND/18-21

21. Filter: Humans

**Search Query for Embase**

1. ‘cerebrovascular accident’/exp

2. Stroke*:ti,ab,kw

3. cerebrovascular:ti,ab,kw

4. ‘brain ischemia’/exp

5. ((brain or vascular or lacunar or venous or cerebral or ischemi*) near/2 (accident* or infarct* or event* or attack*)):ti,ab,kw

6. (cva or cvas):ti,ab,kw

7. ‘nuclear magnetic resonance imaging’/exp

8. (magnetic resonance or MR or MRI or MRIs or MRA or MRDTI):ti,ab,kw

9. (vessel-wall or (vessel wall)):ti,ab,kw

10. (high-resolution or (high resolution)):ti,ab,kw

11. (plaque imaging):ti,ab,kw

12. (vessel near/3 imag*):ti,ab,kw

13. (positive remodeling):ti,ab,kw

14. (brain atherosclerosis):ti,ab,kw

15. (atherosclerotic plaque):ti,ab,kw

16. (intracranial atherosclerosis):ti,ab,kw

17. (intracranial artheriosclerosis):ti,ab,kw

18. (LRNC or ((athero* or steno* or occlus* or ulcer* or plaque* or intraplaque or hemorrhag* or IPH or narrow* or obstruct* or constrict* or bruit* or lipid or fibrous or culprit or lesion*) near/3 (intracranial or cerebr* or brain))):ti,ab,kw

19. or/1-6

20. or/7-8

21. or/9-12

22. or/13-18

23. and/19-22

24. ((animal or nonhuman) not (human and (animal or nonhuman)))/de

25. 23 not 24

**Search Query for The Cochrane Library Wiley**

1 MeSH descriptor: [Stroke] explode all trees

2 stroke

3 cerebrovascular

4 MeSH descriptor: [Brain Ischemia] explode all trees

5 ((brain or vascular or lacunar or venous or cerebral or isch*emi*) near/2 (accident* or infarct* or event* or attack*))

6 cva or cvas

7 MeSH descriptor: [Magnetic Resonance Imaging] explode all trees

8 magnetic resonance or MR or MRI or MRIs or MRA or MRDTI

9 MeSH descriptor: [Contrast Media] this term only

10 Gadolinium or Gd*DTPA or Magnograf or Magnevist or gadopentetate

11. high*resolution

12. vessel *wall

13. plaque imag*

14. MeSH descriptor: [Intracranial Arteriosclerosis] this term only

15. MeSH descriptor: [Plaque, Atherosclerotic] this term only

16. intracranial OR cereb* OR brain

17. (LRNC or ((athero* or steno* or occlus* or ulcer* or plaque* or intraplaque or h*emorrhag* or IPH or narrow* or obstruct* or constrict* or bruit* or lipid or fibrous or culprit or lesion*)

18. OR/1-6

19. OR/7-10

20. OR/11-13

21. OR/14-17

22. AND/18-21

21. Filter: Humans

**II. Supplemental Table. Risk assessment of individual studies using national institute of health quality assessment tool for observational cohort and cross-sectional studies**

| **STUDY** | **Q1** | **Q2** | **Q3** | **Q4** | **Q5** | **Q6** | **Q7** | **Q8** | **Q9** | **Q10** | **Q11** | **Q12** | **Q13** | **Q14** | **Quality Rating** |
| --- | --- | --- | --- | --- | --- | --- | --- | --- | --- | --- | --- | --- | --- | --- | --- |
| Ryu 2009 (2) | + | + | + | - | - | + | + | + | + | + | + | NR | + | - | Fair |
| W Xu 2010 (15) | + | + | + | + | - | + | + | + | + | + | + | NR | + | - | Fair |
| Chung 2012 (13) | + | + | + | + | - | + | + | + | + | - | + | NR | + | - | Fair |
| Kim 2012 (14) | + | + | + | + | - | + | + | + | + | - | - | + | + | - | Fair |
| W Xu 2012 (15) | + | + | + | + | - | + | + | NA | + | - | + | + | + | - | Fair |
| Vakil 2013 (16) | + | + | + | + | - | + | + | + | + | + | + | + | + | + | Good |
| Ryu 2014 (17) | + | + | + | + | - | + | + | + | + | NR | + | NR | + | + | Fair |
| Yang 2014 (18) | + | + | + | + | - | + | + | + | + | + | + | + | + | - | Good |
| Qiao 2014 (19) | + | + | + | + | - | + | + | + | + | + | + | + | + | + | Good |
| Ryoo 2015 (20) | + | + | + | + | - | + | + | + | + | + | + | NR | + | - | Fair |
| P Xu 2015 (21) | + | - | + | + | - | + | + | + | + | - | + | + | + | - | Fair |
| Yu 2015 (22) | + | + | + | + | - | + | + | - | + | - | + | + | + | - | Fair |
| Zhao 2015 (6) | + | + | + | + | - | + | + | + | + | + | + | + | + | + | Good |
| Teng 2016 (23) | + | + | + | + | - | + | + | NA | + | - | + | + | + | + | Good |
| Zhang 2017 (24) | + | + | + | + | - | + | + | + | + | + | + | + | + | - | Good |
| Wang 2017 (25) | + | + | + | + | - | + | + | + | + | - | + | + | + | + | Good |
| Jang 2017 (26) | + | + | + | + | - | + | + | + | + | - | - | + | + | - | Fair |
| Wu 2018 (27) | + | + | + | + | - | + | + | NA | + | - | + | + | + | + | Good |
| Zhu 2018 (28) | + | + | + | + | - | + | + | + | + | + | + | + | + | + | Good |
| Lu 2018 (29) | + | + | + | + | - | + | + | + | + | + | + | + | + | + | Good |

**+**, Yes; **-**, No; *CD, cannot determine; NA, not applicable; NR, not reported

Q1. Was the research question or objective in this paper clearly stated?

Q2. Was the study population clearly specified and defined?

Q3. Was the participation rate of eligible persons at least 50%?

Q4. Were all the subjects selected or recruited from the same or similar populations (including the same time period)? Were inclusion and exclusion criteria for being in the study prespecified and applied uniformly to all participants?

Q5. Was a sample size justification, power description, or variance and effect estimates provided?

Q6. For the analyses in this paper, were the exposure(s) of interest measured prior to the outcome(s) being measured?

Q7. Was the timeframe sufficient so that one could reasonably expect to see an association between exposure and outcome if it existed?

Q8. For exposures that can vary in amount or level, did the study examine different levels of the exposure as related to the outcome (e.g., categories of exposure, or exposure measured as continuous variable)?

Q9. Were the exposure measures (independent variables) clearly defined, valid, reliable, and implemented consistently across all study participants?

Q10. Was the exposure(s) assessed more than once over time?

Q11. Were the outcome measures (dependent variables) clearly defined, valid, reliable, and implemented consistently across all study participants?

Q12. Were the outcome assessors blinded to the exposure status of participants?

Q13. Was loss to follow up after baseline 20% or less?

Q14. Were key potential confounding variables measured and adjusted statistically for their impact on the relationship between exposure(s) and outcome(s)?
